# Similar changes in executive function after moderate resistance training and loadless movement

**DOI:** 10.1371/journal.pone.0212122

**Published:** 2019-02-22

**Authors:** Matthew Vonk, Spencer Wikkerink, Kayla Regan, Laura Elizabeth Middleton

**Affiliations:** Department of Kinesiology, University of Waterloo, Waterloo, Canada; LUNEX International University of Health, Exercise and Sports, LUXEMBOURG

## Abstract

Growing evidence suggests that physical exercise may improve cognitive function in the short- and long-term. Aerobic exercise has been studied most extensively. Preliminary work suggests that resistance training also improves cognitive function, particularly executive function. Conversely, most studies found little dose-effect by intensity. Consequently, cognitive benefits may be elicited, at least in part, by the movement rather than the physical exertion of resistance training. The objective here was to examine and compare acute changes in executive function after resistance training and a loadless movement control among young, healthy adults. Twenty-two young healthy adults (mean age 23.4 years [2.4]; 50% female) completed three conditions, a baseline condition and two experimental conditions (moderate intensity resistance training, loadless movement control). Participants completed a computerized modified Stroop task with concurrent electroencephalography (EEG) before and 10, 20, 30, and 40min after each intervention. Outcomes (incongruent and congruent response time, accuracy, EEG P3 amplitude and latency) were analyzed using mixed linear regression models (factors: condition, time, condition*time). There was a main effect of time for Stroop response time (F_4,84_ = 3.94, p = 0.006 and F_4,84_ = 10.27, p<0.0001 respectively) and incongruent and congruent P3 amplitude (F_4,76_ = 4.40, p = 0.003 and F_4,76_ = 5.09, p = 0.001 respectively). Post-hoc analyses indicated that both incongruent and congruent P3 amplitude were elevated at time points up to and including 40min after the interventions (compared to pre-intervention, p<0.05). Both incongruent and congruent response times were faster at 10min post-intervention than pre-intervention (p<0.04). There was no main effect of condition or interaction between condition and time for either outcome (p≥0.53). Similar improvements in executive function were observed after loadless movement and resistance training, suggesting that movement is at least partially responsible for the benefits to executive function. Future research should continue to probe the influence of movement versus physical exertion in resistance training by including a movement and non-movement control.

## Introduction

Cognitive function refers to mental processes that are involved in acquiring knowledge and understanding through thought and experience. Cognitive function is critical to all voluntary actions, including academic performance, occupational success, and functional independence [1]. Advancing strategies to improve cognitive function could enhance learning and development in early life and reduce the risk of late-life cognitive decline. It is well-established that physical behaviour is importantly influenced by cognitive resources [2,3]. The reverse also seems to be true. Accumulating evidence suggests that cognitive function can be altered by physical behaviour—and, specifically physical exercise [4–6]. Exercise is an appealing approach to enhance cognitive function, in part because of its widespread health and functional benefits [7].

Exercise guidelines for adults recommend both aerobic exercise and resistance training [8–10]. However, most studies of cognitive changes with exercise have investigated the influence of aerobic exercise. There is some evidence that both a single session and a training period of aerobic exercise improves cognitive function among young and older adults [4,11,12], though results are inconsistent. Fewer studies have examined the impact of resistance training on cognitive function (eg. [13,14].

Resistant training has broad benefits to metabolic and musculoskeletal health. Preliminary evidence also suggests that resistance training appears to augment the magnitude of improvements to cognitive function beyond those experienced with aerobic exercise alone [5,14]. Further, resistance training alone for 3 to 24 months may improve executive function, selective attention, and goal planning among older adults compared to a stretching and toning control group [15–18]. However, these training studies offer little evidence for a dose-effect by either resistance training intensity or frequency [15,16]. As a result, it is unclear whether it is the physical exertion required by resistance training that drives the cognitive changes or whether cognitive changes may be partially driven by the novelty of the intervention or the movement itself.

Recent studies suggest that cognitive function may also improve acutely after a single bout of resistance training, though results are variable across studies [13,19–27]. Benefits appear to be greatest and most consistently observed for elements of executive function, including working memory, planning, and inhibitory control [13,19–27]. Executive function refers to control of mental processes to facilitate current goals. In particular, inhibitory control, a component of executive function that involves the suppression or rejection of irrelevant stimuli to achieve a goal-related behavior [28,29], was observed to improve after resistance training when measured with a Stroop or Flanker task (eg. [23,26]).

To leverage the acute cognitive benefits of resistance training, identification of the optimal dose is of interest. One study of the acute cognitive benefits of resistance training compared cognition after rest, low, moderate, and high intensity resistance training. For information processing, there was a linear relationship between resistance training intensity and cognitive improvements, where higher intensity resulted in greater improvements in information processing [21]. However, the relationship between resistance training intensity and executive function (measured with a Stroop task) was quadratic rather than linear. That is, executive function improved after all resistance training intensities compared to rest but improvements were relatively similar across resistance training intensities [21]. This parallels the results from long-term resistance training studies, which show little to no dose-effects of resistance training on high-level cognitive functions [15,16]. As a result, it remains unclear whether the short-and long-term benefits of resistance training on executive function depend on intensity. If not, it is possible that the movement required by resistance training is at least partially responsible for the benefits to executive function. Mechanistically, even loadless movement, if novel, could alter arousal and, thereby, executive function in the short-term [30].

Use of electroencephalography (EEG) may provide insights into the cortical changes that underpin changes in executive function after acute resistance training. Event-related potentials (ERP), in particular, can characterize changes in cortical activity linked to cognitive (or other) stimuli [31]. Although a number of studies have characterized changes in ERP with aerobic exercise [32–34], only one study has used ERP to characterize changes following acute resistance training [26]. The study probed the effects of moderate and high intensity resistance training among healthy young adults on executive function and associated cortical activity (P3 waveform) during a modified Erickson Flanker task [26]. The P3 waveform is one of the most widely studied ERPs of the scalp. It has a centro-parietal distribution and is thought to reflect context and memory updating processes that occurs each time new sensory information is presented and a response is selected. The P3 amplitude appears to depend on capacity to process relevant stimuli while the P3 latency is thought to reflect processing speed [31,35,36]. Greater P3 amplitudes were similarly observed after both moderate and high intensity acute resistance training [26], which is thought to reflect the attentional resources dedicated to the stimuli [31,32].

The objective of this study was to examine and compare the acute effects of moderate intensity resistance training and loadless movement on executive function among young, healthy adults. A secondary objective was to understand the time course of effects by assessing executive function at several time points up to 40min post-intervention. Prior studies have primarily examined changes in executive function immediately post-exercise or when heart rate returns near baseline (approximately 40min post-exercise) [4,25,32]. We hypothesized that executive function would improve after both resistance training and loadless movement, but that improvements would be greater after resistance training. These investigations will provide insight into whether the physical exertion of resistance training provides additional benefit to executive function over loadless movement.

## Material and methods

### Participants

Twenty-two young healthy adults (aged 20–30 years) were recruited for this study. Participants were screened for readiness to exercise using the Physical Activity Readiness Questionnaire (PAR-Q) [37]. Participants who had musculoskeletal disorders that would interfere with resistance training, had concussion(s) in the last year, had epilepsy, or were taking medications that would alter heart rate or blood pressure were excluded. This study was reviewed and received ethics clearance by a University of Waterloo research ethics board. All participants provided written informed consent.

### Study design

This study used a repeated measures design to investigate and compare the effects of moderate intensity resistance training and loadless movement on executive function. Each participant completed three sessions, a familiarization session and two experimental sessions. The sessions were completed two weeks apart on the same day of the week and at the same time of day. The order of the two experimental conditions was randomized. All participants were asked to refrain from exercise and consumption of stimulants and depressants (e.g., caffeine, ephedrine, or tetrahydrocannabinol) on the days of testing, which was confirmed upon arrival to the lab.

#### Baseline protocol

In the baseline session, participants first reported demographic information and had height and weight measured. They next reported their past week of physical activity using the International Physical Activity Questionnaire (IPAQ) and completed a resistance training questionnaire that probed resistance training frequency, duration, and intensity over the last 6 months.

Participants then practiced the assessment protocol, which familiarized them to study protocol, a modified Stroop task, and EEG set up and collection. Exposure to novel experiences including experimental procedures may alter arousal and, thereby, influence cognitive performance. Participants completed 3 blocks of 100 trials of the Stroop task (detailed below), sat for quiet rest for 30 minutes, and then completed two blocks of the Stroop task 4 more times, simulating the timing of the assessment protocol.

Participants then completed their 10-RM assessment for each of 5 resistance training exercises (leg press, pull-down, hamstring curl, vertical chest press, bilateral bicep curl, bilateral tricep extension) in accordance with the 10-RM testing procedure of the National Strength and Conditioning Association [9]. The 10-RMs were used to determine the loads for the subsequent resistance training condition. In brief, participants performed 4 to 5 sets of each exercise at increasing weight with 2 to 3 minutes rest between sets. The increment of weight increase was dependent on the difficulty of the prior set, as per National Strength and Conditioning Association testing protocol [9]. The 10-RM was identified from the maximal weight at which the participant could perform the exercise for no more than 10 repetitions. If a participant could not complete the full ten repetitions before fatigue, a repetition scheme calculator was used to estimate their 10-RM [38].

After completing the 10-RM testing, participants were randomized to the order of the subsequent two experimental conditions.

#### Experimental protocol

Upon arrival, the EEG cap was set up, as described below. Next, participants completed 3 blocks of 100 trials of the Stroop task. Participants then proceeded to the training area [approximately 5min delay).

**Fig 1:**
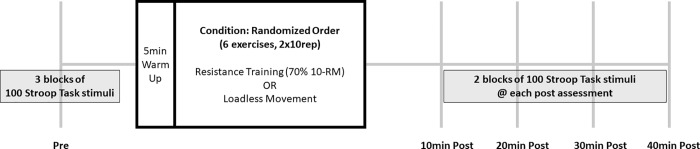
Schema of the experimental protocol.

**Fig 1** shows the experimental schema. Each intervention took approximately 30min. The two interventions were similar except for the intensity of training. Both interventions started with a 5min warm-up. The warm up consisted of exercising on a stationary bike ergometer for 3.5min and then performing 10 jumping jacks and 10 band pull-apart three times. After the warm up, participants performed two consecutive sets of 10 repetitions for each exercise with 60s of rest between sets and 90s of rest between exercises. During the resistance training condition, participants lifted weights corresponding to 70% of their 10-RM for each exercise, which aligns with moderate intensity [9]. This intensity was chosen because moderate intensity resistance training has been used most often in prior research to garner cognitive benefits [9,13,14,21,23].

During the loadless movement condition, participants performed the movements with the lowest weight possible on each exercise machine.

After completion of the intervention, participants returned to the assessment area where impedances of the EEG were checked, which took approximately 8 minutes. Participants then completed two blocks of 100 trials of the Stroop task at 10, 20, 30, and 40min post-intervention. Each block of 100 trials took approximately 3 minutes to complete. There was a 1-minute break between the two blocks.

Heart rate was measured using a Polar heart rate monitor and was recorded after each set during the resistance training and loadless movement conditions as well as 5min before, 30, and 50min after the intervention. Rating of Perceived Exertion (RPE) was also reported at the same intervals using the 6–20 scale to confirm intensity of effort [39].

### Measures

#### Stroop task

A computerized modified Stroop task was used to probe executive function [20,21]. The Stroop stimulus consisted of a written name of a color (red, blue, green, or yellow) printed in one of four colors (same colors as the words), displayed on a monitor. The participant responded with one button if the stimulus was congruent (word written in the color that matches its meaning) and a different button if the stimulus was incongruent (word written in a different color which does not match its meaning). The ratio of congruent to incongruent stimuli was 3:1 within each 100 trial block, which has been shown to magnify the Stroop effect compared to other ratios [40]. The Stroop effect describes the slowing and diminished accuracy of responses to incongruent trials compared to congruent trials [40,41]. The Stroop task was generated and delivered electronically using Stim2 software [42].

Participants sat 185cm away from a 40-inch computer monitor. Participants were instructed to look at a small white fixation-cross presented in the middle of a black screen where the target stimuli appeared and to respond as quickly as possible to the stimulus when it appeared. The response pad was placed on a table on their dominant side. Participants responded with their index for incongruent stimuli and their middle finger for congruent stimuli. The stimulus was 5cm high and 10 to 15cm wide (depending on the word presented). Each stimulus was displayed for 150ms with a 1000ms response window. There was a 2000ms inter-trial duration. A minimum response time of 250ms was required for correct responses to eliminate anticipatory responses.

Accuracy and response time were collected using the Stim2 software. Trials with errors or with no registered response within the 1000ms window were considered incorrect. Response time was only considered for correct trials. Tasks were performed in a dark room with dividers on both sides to reduce distraction and horizontal eye movement. Participants wore moldable earplugs and over-the-ear headphones to reduce auditory noise during the tasks.

#### Electroencephalography recording

Cognitive control was assessed using a computerized Stroop task with concurrent monitoring of EEG to capture the P3 waveform. EEG was recorded during the Stroop task using a 64-channel Quik Cap [42]. The EEG signal was processed at the Pz electrode, as identified in the International 10–20 system. Electrodes were also placed above and below the left eye and lateral to both eyes to create an electrooculogram (EOG) to capture blinks and eye movements to aid in artifact detection. Electrodes on the mastoids were collected as reference electrodes. The impedances for each electrode were less than 5 kΩ. The EEG signal was sampled at 500 Hz.

### Analysis

#### Electroencephalography analysis

EEG data was analyzed using the Curry Neuroimaging Suite 7.0.9 and 7.0.10SB software [42]. The EEG signal was filtered with a high pass filter of 0.5 Hz and a low pass filter of 30 Hz. Next, the signal was referenced to the mastoids and epoched from 100ms prior to stimulus onset to 1000ms post-stimulus. Post-stimulus signal was baseline corrected to the pre-stimulus interval. A covariance regression reduction method was used to subtract artifact contamination due to blinks and other artifacts [43]. Each epoch was then visually assessed for excessive noise and artifacts. If artifacts were still present within the P3 window, the epoch was rejected from the analysis. Trials with response errors were also rejected. The remaining trials were averaged.

The P3 amplitude of the averaged epoch was defined as the most positive peak occurring 350 to 750ms after stimulus presentation (μV). P3 latency was defined as the time in ms at which this maximal positive peak occurred [31].

#### Statistical analysis

Participant and exercise characteristics are presented as mean and standard deviations or percent (n), as appropriate. Differences in exercise characteristics across conditions were determined using a linear mixed model with a main effect of condition and random effect by participant.

Outcomes (P3 amplitude, P3 latency, Stroop response times and Stroop accuracy) were evaluated using linear mixed models with a random effect for participants. Factors included condition (2-level: loadless movement and resistance training) and time (5-level: Pre and 10, 20, 30, 40min post) and condition x time. Analyses for incongruent and congruent conditions were conducted separately. We analyzed incongruent and congruent conditions separately since we used a 3:1 congruent to incongruent ratio. As a result, incongruent results would have been under-represented in combined analyses. However, a Stroop effect was confirmed for all measurements (F_1,18/20_>58, p<0.0001). Post hoc analyses of significant main or interaction effects were performed using Tukey’s test. A significance level of p = 0.05 was used for all analyses. Effect sizes (partial eta-squared, η_*p*_^2^) were calculated for each F-statistic using recommended methods [44]. Statistical analyses were run with SAS 9.4.

### Results

#### Participant characteristics

Twenty-two young healthy adults completed the study. Participants had an average age of 23 years (SD = 2, range: 20–30 years) and 50% (11) were female. Participant characteristics are displayed in **Table 1**. All participants completed the study and had complete data for the Stroop task. Two participants (1 male, 1 female) were removed from EEG analyses due to technical problems with the data, leaving 20 people with complete data.

**Table 1:** Participant characteristics (n = 22).

Characteristics	Mean ± SD or % (n)
**Age (years)**	23 ± 2
**Sex (% females)**	50% (11)
**Education (years)**	18 ± 12
**Handedness (% right)**	95% (21)
**Resting HR (bpm)**	68 ± 8
**IPAQ (Mets-min/wk)** **IPAQ High Physical Activity**	3931 ± 3065 68% (15)
**Current resistance training (% yes)**	80% (16)
**Any resistance training experience (% yes)**	95% (21)

### Exercise characteristics

Characteristics of participants during the exercise condition are displayed in **Table 2**. The exercise intensity (load) was significantly different between conditions (F_1,21_ = 6703.79, p<0.0001), as expected. HR and RPE during the resistance training intervention were also significantly different than during the loadless movement control condition (F_1,21_ = 451.81, p<0.0001 and F_1,21_ = 497.92, p<0.0001, respectively), as was heart rate immediately post-intervention (F_2,42_ = 18.91, p<0.0001).

**Table 2:** Exercise characteristics. Intensity measures by condition (loadless movement vs resistance training) (mean ± SD or % (n)).

Characteristic	Loadless Movement	Resistance Training	p-for-difference
**% of 10-RM**	8 ± 4	70 ± 1	<0.0001
**Intervention RPE**	7 ± 1	14 ± 1	<0.0001
**Pre-exercise HR**	72 ± 9	70 ± 8	0.38
**Intervention HR**	92 ±12	120 ± 13	<0.0001
**Immediately Post HR**	70 ± 9	78 ± 10	<0.0001
**30min Post HR**	72 ± 10	76 ± 9	0.002
**50min Post HR**	71 ± 9	75 ± 10	0.03

### Stroop task

#### Response times

**Fig 2:**
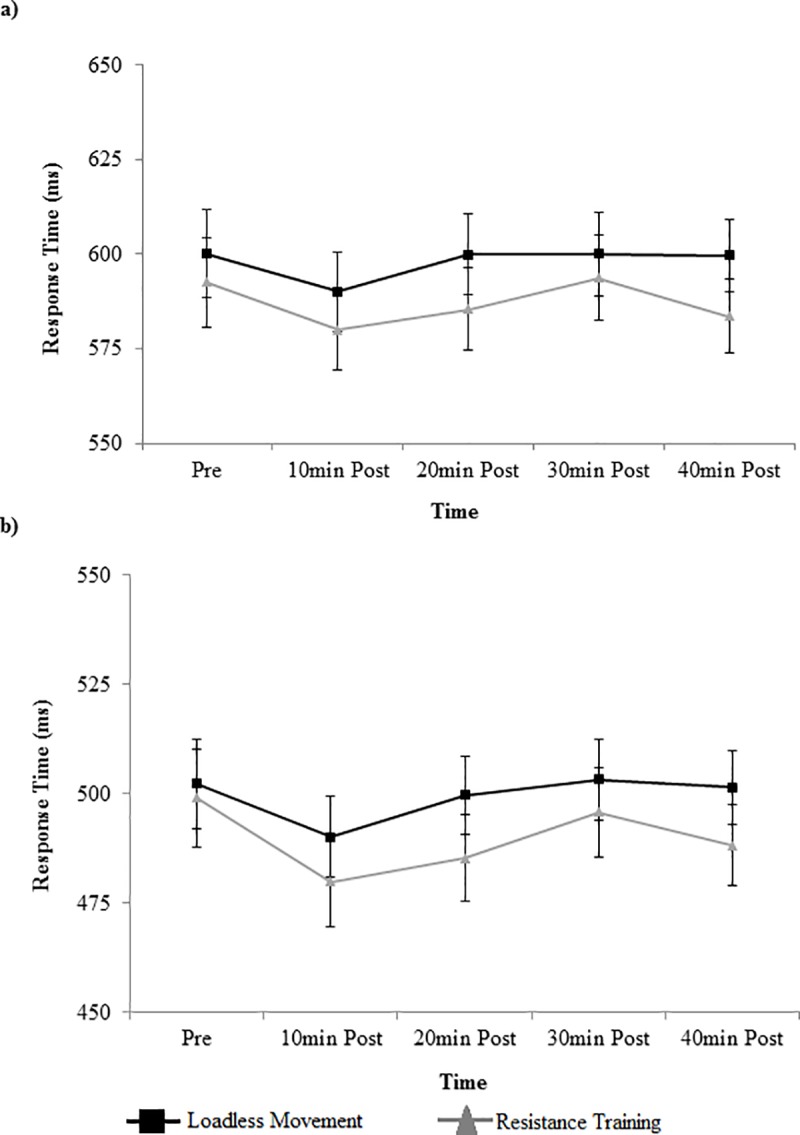
Stroop accuracy. Stroop accuracy (mean±SE) by time, condition, and congruency for: (a) incongruent trials; and (b) congruent trials.

Stroop response times by condition, congruency, and time are displayed in Fig 2. There was a main effect of time for both incongruent and congruent trials (F_4,84_ = 3.94, p = 0.006, η_*p*_^2^ = 0.16 for incongruent; F_4,84_ = 10.27, p<0.0001, η_*p*_^2^ = 0.33 for congruent). For incongruent trials, post-hoc analyses indicated that response times were faster 10min after the interventions (585.0±9.8) than pre-intervention (596.4±10.3) (p = 0.04). For congruent trials, post-hoc analyses indicated that response times were faster 10min after intervention (485.0± 8.7) than at either pre-intervention (502.2± 9.8) (p = 0.0001) or 30min post-intervention (499.5± 8.6) (p = 0.0044). There was no main effect of condition (F_1,21_ = 0.68, p = 0.42, η_*p*_^2^ = 0.03 for incongruent; F_1,21_ = 0.71, p = 0.41, η_*p*_^2^ = 0.03 for congruent) or condition x time interaction effect (F_4,84_ = 0.96, p = 0.43, η_*p*_^2^ = 0.04 for incongruent; F_4,84_ = 1.45, p = 0.22, η_*p*_^2^ = 0.06 for congruent) for either congruency.

#### Accuracy

**Fig 3:**
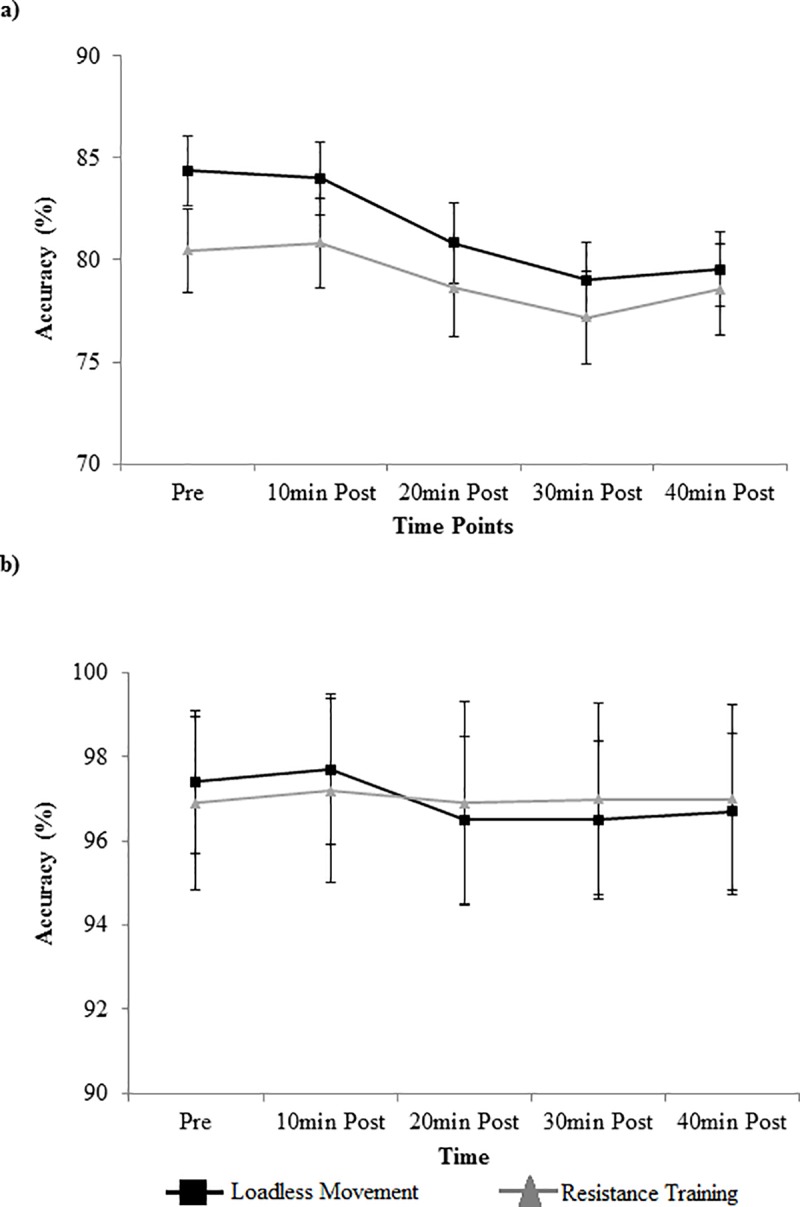
Stroop response times. Stroop response times (mean±SE) by time, condition, and congruency for: (a) incongruent trials; and (b) congruent trials.

Stroop response accuracies by condition, congruency, and time are displayed in Fig 3. Analyses revealed a main effect of time for incongruent trials only (F_4,84_ = 2.83, p = 0.03, η_*p*_^2^ = 0.12). Post-hoc analyses indicated that accuracy was significantly higher for incongruent trials pre intervention (83.4±2.0) compared to 30min post-intervention (78.1±1.9) (p = 0.023). There was no effect of condition (F_1,21_ = 1.33, p = 0.26, η_*p*_^2^ = 0.06) or condition x time interaction effect (F_4,84_ = 0.63, p = 0.64, η_*p*_^2^ = 0.03) for incongruent trials. For congruent trials, there was no effect of condition (F_1,21_ = 0.75, p = 0.41, η_*p*_^2^ = 0.03), time (F_4,84_ = 1.55, p = 0.19, η_*p*_^2^ = 0.07), or condition x time interaction effect (F_4,84_ = 0.45, p = 0.74, η_*p*_^2^ = 0.02) on Stroop task accuracy.

### EEG

**Fig 4:**
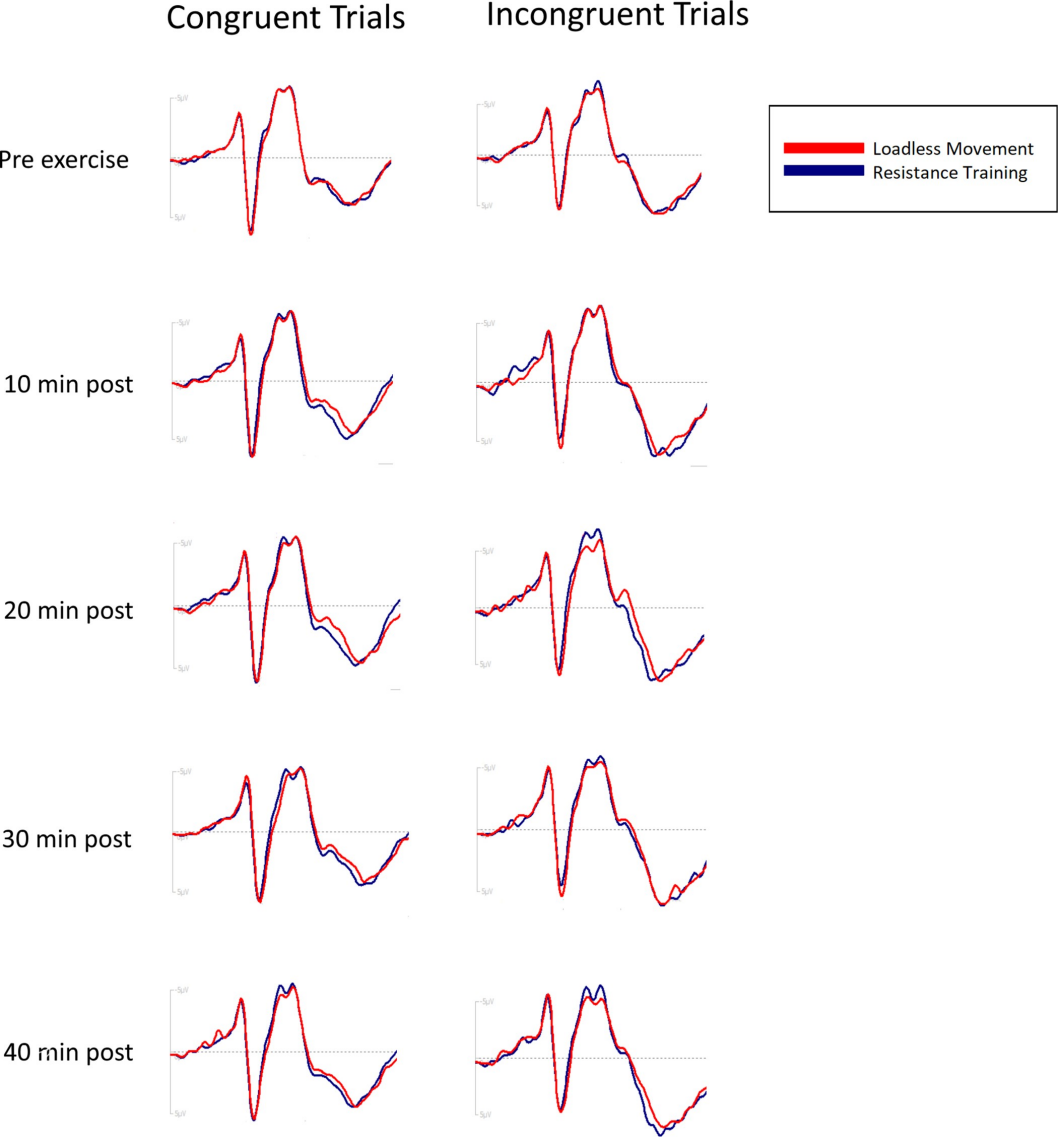
Grand averaged ERP. Grand averaged ERP waveforms at the Pz electrode by congruency.

The grand averaged ERP waveforms by intervention, time point, and congruency are shown in Fig 4.

#### P3 amplitude

P3 amplitudes by condition, congruency, and time are displayed in **Table 3**. Analyses of incongruent and congruent trials revealed a main effect of time for both trial types (F_4,76_ = 4.40, p = 0.003, η_*p*_^2^ = 0.18 for incongruent trials; F_4,76_ = 5.09, p = 0.001, η_*p*_^2^ = 0.21 for congruent trials). Post-hoc analyses of incongruent trials indicated that P3 amplitude was larger at 30min (14.4±0.7) and 40min (14.6±0.7) after the interventions compared to pre-intervention (13.0±0.6) (p<0.011). Post-hoc analyses of congruent trials indicated that P3 amplitude was larger at 10min (10.1±0.4) and 40min (10.2±0.4) after the interventions than pre-intervention (9.3±0.4) (p-for-difference<0.05). There were no effects of condition (F_1,19_ = 0.02, p = 0.89, η_*p*_^2^ = 0.05 for incongruent; F_1,19_ = 0.001, p = 0.95, η_*p*_^2^ = 5.3*10^−5^ for congruent trials) or condition x time interaction effect (F_4,76_ = 2.45, p = 0.05, η_*p*_^2^ = 0.11 for incongruent; F_4,76_ = 1.37, p = 0.25, η_*p*_^2^ = 0.07 for congruent trials) for either congruency.

**Table 3:** P3 amplitudes (μV) for incongruent and congruent trials by condition and time.

Time	Loadless Movement	Resistance training
**Incongruent**		
**Pre**	12.9 ± 0.8	13.2 ± 0.8
**10min Post**	13.6 ± 0.7	14.4 ±0.7
**20min post**	14.8 ± 0.9	13.6 ± 0.9
**30min post**	14.2 ± 0.9*	14.6 ± 0.9*
**40min post**	14.4 ± 0.8*	14.9 ± 0.8*
**Congruent**		
**Pre**	9.3 ± 0.6	9.1 ± 0.7
**10min Post**	9.8 ± 0.6*	10.2 ± 0.7*
**20min post**	9.7 ± 0.7	9.9 ± 0.8
**30min post**	9.7 ± 0.7	9.7 ± 0.8
**40min post**	10.4 ± 0.5*	9.9 ± 0.7*

*Significantly different from pre, p<0.05.

#### P3 latency

P3 latencies by condition, congruency, and time are displayed in **Table 4.** There were no significant effect by condition (F_1,19_ = 2.10, p = 0.16, η_*p*_^2^ = 0.10 for incongruent; F_1,19_ = 0.05, p = 0.97, η_*p*_^2^ = 0.002 for congruent), time (F_4,76_ = 0.75, p = 0.59, η_*p*_^2^ = 0.04 for incongruent; F_4,76_ = 1.29, p = 0.28, η_*p*_^2^ = 0.06 for congruent), or condition x time interaction effect (F_4,76_ = 2.10, p = 0.089, η_*p*_^2^ = 0.10 for incongruent; `F_4,76_ = 0.45, p = 0.77, η_*p*_^2^ = 0.02 for congruent) for either congruency.

**Table 4:** P3 latencies (ms) for incongruent and congruent trials by condition and time.

Time	Loadless Movement	Resistance training
**Incongruent**		
**Pre**	575.4 ± 14.0	556.7 ± 15.8
**10min Post**	565.4 ± 15.2	544.5 ±17.1
**20min post**	553.7 ± 14.2	560.6 ± 15.9
**30min post**	592.4 ± 16.0	548.9 ± 18.0
**40min post**	565.0 ± 19.0	529.7 ± 21.3
**Congruent**		
**Pre**	474.3 ± 18.0	480.6 ± 16.1
**10min Post**	482.3 ± 19.3	475.0 ± 17.1
**20min post**	470.9 ± 21.2	479.8 ± 18.8
**30min post**	466.3 ± 20.8	453.8 ± 18.6
**40min post**	499.3 ± 19.1	481.2 ± 17.1

## Discussion

This study investigated and compared the acute effects of moderate intensity resistance training and loadless movement on executive function. Our results suggest that both resistance training and loadless movement elicited some improvements in executive function, as indicated by faster response times and greater P3 amplitude during a modified Stroop task. However, in contrast to our hypothesis, changes in executive function after exercise were similar between resistance training and loadless movement conditions. This preliminary study suggests that movement may account for at least some of the improvement in executive function observed after resistance training, though this possibility needs to be further explored.

Resistance training is part of physical activity recommendations with well-documented benefits to metabolic and musculoskeletal health [8,10]. Emerging research also suggests that resistance training, when performed acutely or over a period of time, is associated with improvements in cognitive function, and especially executive function [4,23,26].This was the first study to examine the effects of resistance training on executive function relative to an active control (loadless movement) rather than a passive control (rest). In our study, changes in executive function were similar after moderate intensity resistance training and loadless movement. This is in line with one prior study that observed minimal dose-related changes in executive function after light, moderate, and high intensity resistance training [21]. Another study also found similar improvements in executive function and associated cortical processing after moderate and high intensity resistance training [26]. Together, these results suggest that resistance training may improve executive function, but that movement may account at least partially for its effects [21]. In contrast, the magnitude of changes in processing speed was linearly related to exercise intensity in one study [21]. Future work should compare the influence of resistance training, training intensity, and loadless movement across cognitive domains. It is possible that the influence of physical exertion during resistance training varies by cognitive task or domains, as is the case with aerobic exercise [4,8,10,13].

In this study, there was evidence for improvements in executive function between 10 and 30 minutes after resistance training and loadless movement conditions. This is in line with most prior studies, which observed better executive function after resistance training compared before resistance training or compared to a rest condition [13,19–24,26,27]. While one study showed no change in executive function after resistance training, it used exhaustive training (very high intensity [80% of 1-RM] and high repetitions [8–12 repetitions]) [25]. It may be that exhaustive exercise overcomes any improvements induced by movement or low to moderate resistance training. Even if resistance training does not require significant physical exertion to induce improvement in executive function, it may be that exhaustive resistance training raises cortisol levels sufficiently to induce neural noise [45–47], disrupting attentional processing and counteracting the beneficial effects of movement. Similarly, very high intensity or exhaustive aerobic exercise also shows null or negative effects to executive function [33,48].

This was the first study to examine the effects of resistance training (and movement) over time after the intervention, where other studies only had one post-intervention assessment. In this study, post-intervention assessments were performed at 10min intervals up to 40min post-intervention. Improvements in executive function as indicated by Stroop task response times were observed only at 10min after the interventions but changes in P3 amplitude were observed up to 40mins post-intervention. This is in line with literature regarding aerobic exercise, which has observed changes up to 30 minutes after exercise [4,25,32].

In contrast to improvements in Stroop task incongruent response times that occurred only shortly after the interventions, improvements in P3 amplitude peaked 30 to 40min post-intervention. Why P3 amplitude peaks so late after exercise in contrast to response time is unclear. No prior studies of either aerobic exercise or resistance training have characterized ERP associated with cognitive assessments at multiple timepoints post-exercise. It may be that improvements in stimulus classification (as reflected by the P3) peak later than the overall combination of stimulus classification and motor performance (as reflected by response time). Indeed, one study observed greater P3 amplitudes but no change in response accuracy when measured 48minutes after acute aerobic exercise (when heart rates were within 10% of baseline),[32] in line with the present study’s findings. Future studies should use EMG to separate reaction time versus movement time to examine effects by stimulus processing, reaction time, and movement time.

Resistance training is only one part of the recommended physical activity guidelines [8,10]. Aerobic exercise is also recommended and has been examined more extensively relative to cognition in both acute and longitudinal interventions [4,11]. Though results are variable, a recent meta-analysis found that there were immediate and delayed improvements in cognitive function, and executive function specifically, after aerobic exercise [4]. In one study, Stroop task performance improved after acute aerobic exercise, which was associated with increased activation in the dorsolateral prefrontal cortex measured with functional near infrared spectroscopy (fNIRS) [49]. Whether this occurs with resistance training or loadless movement is unclear.

A few studies have compared the effects of resistance training and aerobic exercise with mixed results. One study showed that acute aerobic exercise improve cognitive function but acute resistance training did not [25], while another showed that both types of exercise improved cognitive function compared to a resting control [27]. Doing both may be most beneficial as a meta-analysis indicated that aerobic exercise training interventions that were paired with resistance training were associated had greater cognitive benefits than those that were not [5].

Our study has several strengths. It was the first to use an active control to evaluate the effects of resistance training on executive function. In addition, this study was the first to measure cognitive changes after resistance training over an extended period after exercise to better understand the time course of effects. However, the study also has limitations. Though this study has an active control, we did not include a passive control (rest). Therefore, we cannot definitively conclude that either resistance training or loadless movement improves executive function. Further, it is possible that we were underpowered to detect differences between resistance training and this active control, though we estimated our sample size required based on a recent study [26]. Also, it is possible that participants experienced boredom or mental fatigue due to the extended testing period post-intervention, which may have influenced our findings at later time points. Alternatively, it is possible that the repeated testing induced learning effects. However, this is unlikely as participants had substantial practice of the Stroop task during the baseline session. In addition, our study used a computerized modified Stroop task to assess inhibitory control. Although this assessment allowed us to carefully lock EEG measures to the Stroop task, the assessment had some important differences from the standard Stroop task. Participants had to determine the congruency of the stimuli rather than saying the color aloud. This may have required less executive function [50]. Even so, the response times are considerably longer than those typically observed with a Flanker task (for example, [25]), suggesting significant cognitive demand. In addition, our sample was composed of young, fit, healthy adults, most of whom participated regularly in resistance training (80%). It is possible that the effects of resistance training versus movement may be different in less trained populations or in older adults, as used in prior studies [13,23,25,26]. Finally, the loadless movement condition used the absolute lowest setting for the exercises, meaning there remained some very minimal resistance. We cannot conclude that this very minimal load is not required for benefits to executive function.

Our results suggest that minimal physical exertion may be required to elicit improvements in executive function after resistance training. Future research should extend these preliminary investigations to better understand the relative influence of movement and physical exertion on executive function and other cognitive functions, as well as explore underlying mechanisms. Optimistically, our results suggest that movement itself may be beneficial to executive function, which may be feasible and practical for a broader range of the population. Given the significant benefits of moderate intensity resistance training to bone and muscle health, however, it may be wise to continue to include physical exertion in a resistance training regimen for brain and body health.

## References

[1] Wessinger C., Clapham E. Cognitive Neuroscience: An Overview. Encylopedia Neurosci. 2009;1117–22.

[2] Holroyd CB, Mcclure SM. Hierarchical Control Over Effortful Behavior by Rodent Medial Frontal Cortex: A Computational Model. Psychol Rev. 2014;122(1):54–83. 10.1037/a0038339 25437491

[3] Nashed JY, Crevecoeur F, Scott SH. Rapid Online Selection between Multiple Motor Plans. J Neurosci. 2014;34(5):1769–80. 10.1523/JNEUROSCI.3063-13.2014 24478359 PMC8186509

[4] Chang YK, Labban JD, Gapin JI, Etnier JL. The effects of acute exercise on cognitive performance: a meta-analysis. Brain Res. 2012 5 9 [cited 2014 May 1];1453(250):87–101. 22480735 10.1016/j.brainres.2012.02.068

[5] Colcombe S, Kramer AF. Fitness effects on the cognitive function of older adults: A Meta-Analytic study. Psychol Sci. 2003 3 [cited 2014 Apr 29];14(2):125–30. 10.1111/1467-9280.t01-1-01430 12661673

[6] Rockwood K, Middleton L. Physical activity and the maintenance of cognitive function. Alzheimer’s Dement. 2007;3(2 SUPPL):38–44. 10.1016/j.jalz.2007.01.003 19595973

[7] Public Health Agency of Canada Physical Activity Benefits. Vol. 54, Public Health Agency of Canada 2014 p. 1.

[8] American college of sports medicine’s guidelines for exercise testing and prescription. Am Coll Sport Med. 2007;(7th ed).

[9] Baechle TR, Earl RW. Essentials of Strength and Conditioning. 2nd ed Human Kinetics Publishing; 2000.

[10] World Health Organization. Global Strategy on Diet, Physical Activity and Health [Internet]. 2014. Available from: http://www.who.int/dietphysicalactivity/factsheet_recommendations/en/

[11] Angevaren M, Aufdemkampe G, Hjj V, Aleman A, Vanhees L. Physical activity and enhanced fitness to improve cognitive function in older people without known cognitive impairment (Review). Cochrane Collab. 2008;(3). 10.1002/14651858.CD005381.pub3 18646126

[12] Smith PJ, Blumenthal JA, Hoffman BM, Strauman TA, Welsh-bohmer K, Jeffrey N, et al Aerobic exercise and neurocognitive performance: a meta- analytic review of randomized controlled trials. Psychosom Med. 2011;72(3):239–52. 10.1097/PSY.0b013e3181d14633 PMC2897704 20223924

[13] Chang Y-K, Ku P-W, Tomporowski PD, Chen F-T, Huang C-C. Effects of acute resistance exercise on late-middle-age adults’ goal planning. Med Sci Sports Exerc. 2012 9 [cited 2014 Nov 26];44(9):1773–9. 10.1249/MSS.0b013e3182574e0b 22460477

[14] de Asteasu MLS, Martínez-Velilla N, Zambom-Ferraresi F, Casas-Herrero Á, Izquierdo M. Role of physical exercise on cognitive function in healthy older adults: A systematic review of randomized clinical trials. Ageing Res Rev. Elsevier B.V.; 2017;37:117–34. 10.1016/j.arr.2017.05.007 28587957

[15] Cassilhas RC, Viana V a R, Grassmann V, Santos RT, Santos RF, Tufik S, et al The impact of resistance exercise on the cognitive function of the elderly. Med Sci Sports Exerc. 2007 8;39(8):1401–7. 10.1249/mss.0b013e318060111f 17762374

[16] Liu-ambrose T, Nagamatsu LS, Graf P, Beattie BL, Ashe MC, Handy TC. Resistance Training and Executive Functions. Arch Intern Med. 2010;170(2):170–8. 10.1001/archinternmed.2009.494 20101012 PMC3448565

[17] Nagamatsu LS, Handy TC, Hsu CL, Voss M, Liu-Ambrose T. Resistance training promotes cognitive and functional brain plasticity in seniors with probable mild cognitive impairment. Arch Intern Med. 2012 4 23;172(8):666–8. 10.1001/archinternmed.2012.379 22529236 PMC3514552

[18] Tsai C-L, Wang C-H, Pan C-Y, Chen F-C. The effects of long-term resistance exercise on the relationship between neurocognitive performance and GH, IGF-1, and homocysteine levels in the elderly. Front Behav Neurosci. 2015;9(February):23. 25713518 10.3389/fnbeh.2015.00023 PMC4322723

[19] Alves CR, Gualano B, Takao PP, Avakian P, Fernandes RM, Morine D, et al Effects of acute physical exercise on executive functions: a comparison between aerobic and strength exercise. J Sport Exerc Psychol. 2012;34(4):539–49. 22889693 10.1123/jsep.34.4.539

[20] Chang Y-K, Etnier JL. Effects of an acute bout of localized resistance exercise on cognitive performance in middle-aged adults: A randomized controlled trial study. Psychol Sport Exerc. 2009 1 [cited 2014 Mar 19];10(1):19–24.

[21] Chang Y-K, Etnier JL, Barella L a. Exploring the relationship between exercise-induced arousal and cognition using fractionated response time. Res Q Exerc Sport. 2009 3;80(1):78–86. 10.1080/02701367.2009.10599532 19408470

[22] Chang Y-K, Chu I-H, Chen F-T, Wang C-C. Dose-response effect of acute resistance exercise on Tower of London in middle-aged adults. J Sport Exerc Psychol. 2011 12;33(6):866–83. 22262709 10.1123/jsep.33.6.866

[23] Chang Y, Tsai C, Huang C, Wang C, Chu I. Effects of acute resistance exercise on cognition in late middle-aged adults: General or specific cognitive improvement? J Sci Med Sport. 2014;(17):51–5. 10.1016/j.jsams.2013.02.007 23491140

[24] Dunsky A, Abu-Rukun M, Tsuk S, Dwolatzky T, Carasso R, Netz Y. The effects of a resistance vs. an aerobic single session on attention and executive functioning in adults. PLoS One. 2017;12(4):1–13. 10.1371/journal.pone.0176092 PMC5404838 28441442

[25] Pontifex MB, Hillman CH, Fernhall B, Thompson KM, Valentini T a. The effect of acute aerobic and resistance exercise on working memory. Med Sci Sports Exerc. 2009 4;41(4):927–34. 10.1249/MSS.0b013e3181907d69 19276839

[26] Tsai C-L, Wang C-H, Pan C-Y, Chen F-C, Huang T-H, Chou F-Y. Executive function and endocrinological responses to acute resistance exercise. Front Behav Neurosci. 2014 1;8(August):262. 25136300 10.3389/fnbeh.2014.00262 PMC4117935

[27] Harveson AT, Hannon JC, Brusseau TA, Podlog L, Papadopoulos C, Durrant LH, et al Acute Effects of 30 Minutes Resistance and Aerobic Exercise on Cognition in a High School Sample. Res Q Exerc Sport. 2016;1367(April):1–7. 10.1080/02701367.2016.1146943 26958898

[28] Hommel B, Ridderinkhof KR, Theeuwes J. Cognitive control of attention and action: issues and trends. Psychol Res. 2002 11 [cited 2014 Oct 7];66(4):215–9. 10.1007/s00426-002-0096-3 12466920

[29] Purves D, Cabeza R, Huettel SA, LaBar KS, Platt MS, Woldorff MG. Principles of Cognitive Neuroscience 2nd ed Sunderland MA: Sinauer Associates Inc; 2013.

[30] Brody H. The Placebo Response: Recent Research and Implications for Family Medicine. J Fam Pract. 2000;49(7):649–54. 10923577

[31] Luck SJ. An Introduction to the Event-Related Potential Technique Vol. 78, Monographs of the Society for Research in Child Development 2005. 388 p.

[32] Hillman CH, Snook EM, Jerome GJ. Acute cardiovascular exercise and executive control function. Int J Psychophysiol. 2003 6;48(3):307–14. 12798990 10.1016/s0167-8760(03)00080-1

[33] Kamijo K, Nishihira Y, Hatta A, Kaneda T, Wasaka T, Kida T, et al Differential influences of exercise intensity on information processing in the central nervous system. Eur J Appl Physiol. 2004 7;92(3):305–11. 10.1007/s00421-004-1097-2 15083372

[34] Kamijo K, Hayashi Y, Sakai T, Yahiro T, Tanaka K. Acute Effects of Aerobic Exercise on Cognitive Function in Older Adults. J Gerontol Psychol Sci. 2009;356–63. 10.1093/geronb/gbp030 19363089

[35] Donchin E, Coles MGH. Is the P300 component a manifestation of context updating? Behav Brain Sci. 1988;11(3):357–74.

[36] Kok A. On the utility of P3 amplitude as a measure of processing capacity. Psychophysiology. 2001;38(3):557–77. 11352145 10.1017/s0048577201990559

[37] Canadian Society Of Exercise Physiology. Pre-Screening for Physical Activity Participation. [Internet]. 2016. Available from: http://www.csep.ca/en/publications/get-active-questionnaire

[38] Nikolov I. Rep Scheme Calculator [Internet]. 2015. Available from: https://ivannikolov.com/calculators/rep-max-calculator/

[39] Borg G. Perceived exertion as an indicator of somatic stress. Scand J Rehabil Med. 1970;2(2):92–8. 5523831

[40] Bélanger S, Belleville S, Gauthier S. Inhibition impairments in Alzheimer’s disease, mild cognitive impairment and healthy aging: effect of congruency proportion in a Stroop task. Neuropsychologia. 2010 1 [;48(2):581–90. 10.1016/j.neuropsychologia.2009.10.021 19879885

[41] Lansbergen MM, Kenemans JL. Stroop interference and the timing of selective response activation. Clin Neurophysiol. 2008 10;119(10):2247–54. 10.1016/j.clinph.2008.07.218 18762447

[42] Compumedics Neuroscan. Curry Neuroimaging Suite 7.0.10SB. 2015.

[43] Moretti D V., Babiloni F, Carducci F, Cincotti F, Remondini E, Rossini PM, et al Computerized processing of EEG-EOG-EMG artifacts for multi-centric studies in EEG oscillations and event-related potentials. Int J Psychophysiol. 2003;47(3):199–216. 12663065 10.1016/s0167-8760(02)00153-8

[44] Lakens D. Calculating and reporting effect sizes to facilitate cumulative science: A practical primer for t-tests and ANOVAs. Front Psychol. 2013;4(NOV):1–12. 10.3389/fpsyg.2013.00001 24324449 PMC3840331

[45] Henckens MJ a G, van Wingen G a, Joëls M, Fernández G. Time-dependent effects of cortisol on selective attention and emotional interference: a functional MRI study. Front Integr Neurosci. 2012 1 [cited 2014 Dec 3];6(August):66. 22973203 10.3389/fnint.2012.00066 PMC3428804

[46] Lambourne K, Tomporowski P. The effect of exercise-induced arousal on cognitive task performance: a meta-regression analysis. Brain Res. 2010 6 23 [cited 2014 Mar 21];1341:12–24. 10.1016/j.brainres.2010.03.091 20381468

[47] McMorris T, Sproule J, Turner A, Hale B. Acute, intermediate intensity exercise, and speed and accuracy in working memory tasks: A meta-analytical comparison of effects. Physiol Behav. Elsevier Inc.; 2011;102(3–4):421–8. 10.1016/j.physbeh.2010.12.007 21163278

[48] Kamijo K, Nishihira Y, Higashiura T, Kuroiwa K. The interactive effect of exercise intensity and task difficulty on human cognitive processing. Int J Psychophysiol. 2007;65:114–21. 10.1016/j.ijpsycho.2007.04.001 17482699

[49] Byun K, Hyodo K, Suwabe K, Ochi G, Sakairi Y, Kato M, et al Positive effect of acute mild exercise on executive function via arousal-related prefrontal activations: An fNIRS study. Neuroimage. Elsevier Inc.; 2014;98:336–45. 10.1016/j.neuroimage.2014.04.067 24799137

[50] Penner Kobel, Stocklin Weber, Opwis Pasquale. The Stroop Task: Comparison Between Original Paradigm and Computerised Version in Children and Adults. Clin Neuropsychol. 2012;26(7):1142–53. 10.1080/13854046.2012.713513 22928670

